# Predictors of Perceived Healthcare Professionals’ Well-Being in Work Design: A Cross-Sectional Study with Multigroup PLS Structural Equation Modeling

**DOI:** 10.3390/healthcare12131277

**Published:** 2024-06-26

**Authors:** Danijela Nesic, Marko Slavkovic, Nebojsa Zdravkovic, Nikola Jerkan

**Affiliations:** 1 Health Center Nis, Vojvode Tankosica 15, 18101 Nis, Serbia; danijela.nesic@domzdravljanis.co.rs (D.N.); nikola.jerkan@domzdravljanis.co.rs (N.J.); 2 Department of Management and Business Administration, Faculty of Economics, University of Kragujevac, Liceja Knezevine Srbije 3, 34000 Kragujevac, Serbia; 3 Faculty of Medical Science, University of Kragujevac, Svetozara Markovica 69, 34000 Kragujevac, Serbia; nzdravkovic@medf.kg.ac.rs

**Keywords:** work design, well-being, overall satisfaction, knowledge characteristics, social characteristics, task characteristics, work context

## Abstract

The growing attention paid to employee well-being in the healthcare system, along with the reinforcement of factors that drive change in the work environment, provides a strong foundation for studying the relationship between work design and its related outcomes. The aim of this study was to examine the relationship between the elements of work design and the overall well-being of healthcare professionals, particularly the differences across multiple groups of various genders and age brackets. A cross-sectional study design was employed, and a convenience sampling method was used. The study participants were healthcare professionals, and a total of 427 valid surveys were collected. The partial least squares structural equation modeling (PLS-SEM) approach was deployed to test the relationship between the determinants of work design and the perceived well-being of healthcare professionals. The results indicate a strong positive relationship between the social characteristics of work and overall well-being, as well as a positive contribution of the work context to the perceived well-being of healthcare professionals. The findings validated that knowledge characteristics had no beneficial impacts on overall well-being; nor did the task characteristics of work design. Although the mentioned associations failed to demonstrate statistical significance, the results nonetheless have significant practical consequences that are comparable to those of the relationships that demonstrated statistical significance.

## 1. Introduction

Work design gained new perspectives and importance during the COVID-19 pandemic in the field of remote work [1,2]. The impact of recent technological advances on the social aspects of organizations provides a sturdy foothold for work design in the digital age, greatly affecting employee well-being [3]. Since organizational tasks are interconnected, work design encompasses decisions regarding the nature of work, techniques, work processes, and resources that employees utilize. Through job structuring, work design considers factors such as required skills, control, task diversity, role clarity, and social support. By integrating these elements, managers aim to increase motivation, facilitate performance improvement, and promote positive mental states and well-being among employees [4,5,6]. Healthcare professionals encounter myriad challenges that can compromise their well-being [7]. These challenges include striving to maintain high-quality healthcare, managing heavy workloads, and working under resource constraints [8,9,10]. Additionally, factors such as long shifts, overtime, and physical and mental fatigue contribute to burnout among healthcare workers, further diminishing their well-being [11,12,13].

As a subjective phenomenon derived from one’s own experience, well-being encompasses happiness and satisfaction, leading to an overall positive experience that is essential for efficiently fulfilling tasks [14], and it is a result of the subjective perception of an individual’s quality of life [15]. Well-being is a double-edged term within the healthcare system, as it can be applied to both patients [16] and healthcare professionals [17]. The concept of well-being is multifaceted, as it includes physical well-being, which pertains to aspects such as good health; mental well-being, which is associated with happiness; spiritual well-being, which refers to the state of mind, spirit, and emotions; and social well-being. Additionally, cultural well-being is significant, as it relates to the ability to express one’s own culture, norms, and traditions [18,19,20]. Our study affirms the multidimensional character of well-being and examines this concept by measuring the overall level of satisfaction with the various aspects of the quality of personal life, including the aforementioned dimensions, with the exception of cultural well-being. In healthcare organizations, fostering well-being involves implementing flexible working arrangements to mitigate frustration, providing training, coaching, development programs, and emotional counseling, and offering healthy food options [21].

Human resource practices in organizations, such as those related to work design, can affect employee well-being [22]. Prior research established an association between various aspects of work design and the well-being of employees. The findings revealed that a significant percentage of the variation in employee well-being may be attributed to various work characteristics [23]. An international study revealed that work meaningfulness and social support are substantial predictors of well-being [24], underscoring the significance of task characteristics and social characteristics in work design. The results of the meta-analysis showed that job crafting, which refers to adjustments in the tasks performed by individuals and the relationships established between employees, has a positive impact on employee well-being [25]. A similar study showed that the employees who had the highest level of well-being engaged in the most job crafting and reported the greatest amount of support for their autonomy [26]. This indicates that job redesign in the context of task characteristics is a viable approach to regulating the well-being of individuals. Additionally, the development of social relationships among employees demonstrates a favorable perspective for enhancing well-being, thereby validating the important role of social characteristics in work design. 

A comprehensive well-being program for healthcare professionals should consider the heightened occupational hazards that they face [27]. In addition, working conditions have been identified as a factor that can significantly support overall well-being [28]. Specific risks in this sector, such as the risk of needle-stick injuries, cuts, or infection, significantly increase stress and affect the well-being of healthcare professionals. Therefore, the work context, which encompasses a variety of aspects of the work environment, is an important element of work design that can have a dual impact on well-being. Firstly, it can reduce the risk of injury and health hazards, and secondly, it can foster a positive experience while working in a pleasant environment. 

The job demand–resource theory [29] offers a solid foundation for comprehending the relationship between the knowledge characteristics of work design and the well-being of employees. Job demands and job resources are the two primary categories of each job, and their alignment predicts well-being [30] according to the aforementioned theory. Job resources, such as social support and skill variety, are the components of work that assist employees in managing job demands and completing their objectives. When job resources are not sufficient to meet job demands, motivation and job performance decrease, creating a negative impact on work-related well-being. Empirical data have indicated that work-related well-being has a significant impact on well-being in life overall [31]. Through the aforementioned mechanism, high job demands in the domain of the knowledge and skills needed to perform work lead to a negative impact on well-being. Therefore, the knowledge characteristics of work design appear to be an important predictor of employees’ well-being.

Effective work design should include both intrinsic and extrinsic factors that contribute to well-being. For healthcare professionals, intrinsic factors include a sense of achievement, the clear communication of goals, autonomy, responsibility, opportunities for learning, and knowledge sharing. Extrinsic factors often involve working hours, the level of control, job security, opportunities for promotion, and wages [19]. Ensuring well-being within healthcare organizations necessitates the clear specification of job demands, requisite resources, and the dynamics of employee relationships. Work design should consider leadership structures, levels of employee autonomy, the span of responsibilities, and the factors contributing to motivation and satisfaction [21]. Effective job design should cater to employees’ innate needs for creativity, continual learning, social engagement, and knowledge sharing [32]. Going beyond ergonomic considerations [5], strategic workplace design addresses employees’ articulated needs, ensuring that tasks possess clarity, significance, autonomy, and skill variety and that constructive feedback is offered [33,34,35].

Tasks enriched with autonomy, social capital, skill variety, and participation promote well-being [36]. Incorporating elements such as decision making, career planning, learning opportunities, human capital development, and knowledge sharing into job design enhances well-being and job satisfaction [37]. Health organizations enhance well-being by involving employees in training and knowledge development programs and fostering creative and innovative thinking [38,39]. Within healthcare organizations, work design should facilitate flexible work, provide technical equipment, and establish effective communication systems for exchanging relevant information [8]. Considering the above, well-being and job satisfaction necessitate that work design is aimed not only at maximizing performance but also at socio-technical factors that positively influence employees’ emotions, physical state, and mental state [4,40]. 

In summary, the purpose of this study is to assess the significance of the relationships between work context, task characteristics, knowledge characteristics, and the social characteristics of work design in healthcare organizations and the perceived well-being of employees. Consequently, the aim of this study is to explore the impact of work design factors on the perceived well-being of healthcare professionals.

## 2. Materials and Methods

### 2.1. Sampling Methods and Procedure

Healthcare professionals were identified as key informants in this study, and a convenience sampling method was used to collect primary data. We decided to employ a cross-sectional study design for the purposes of this research. The study participants were healthcare professionals employed in healthcare organizations in Central and Southern Serbia. Prior to contact with the potential respondents, the verbal permission of the management of each healthcare organization was obtained. The sampling procedure adhered to the principles outlined in the Declaration of Helsinki, including its latest amendments, to ensure compliance with ethical standards. The study was approved by the Council of the Faculty of Medical Science, University of Kragujevac (protocol code 01-15323/3 from 29 December 2023). The first page of the questionnaire explicitly included a statement of informed written consent. Initially, the potential respondents were provided with information on the academic objective of the study and were guaranteed anonymity. The questionnaire did not include inquiries pertaining to highly sensitive personal information. In addition, all the participants were guaranteed that their demographic data would only be used for statistical analysis and would be securely protected from any access by other entities. The respondents were given the autonomy to decide whether to participate according to their availability and were encouraged to reach out with any additional questions or clarifications regarding the questionnaire if needed. 

The survey was carried out between January and February 2024. The approach yielded a total sample size of 427 healthcare professionals, and their characteristics are outlined in Table 1. In summary, the sample predominantly consisted of females, with a majority of individuals being under 40 years old. Most of the healthcare professionals possessed a high school degree or high school diploma, while a smaller portion held a graduate degree or higher.

### 2.2. Survey Instrument and Measurement

A structured questionnaire that was divided into three sections was used as an essential prerequisite to collect primary data and carry out the intended statistical analyses. The first part focused on measuring the work design elements, which were noted as the independent variables and comprised four constructs: job characteristics, knowledge characteristics, social characteristics, and work context. These constructs were measured with the Work Design Questionnaire (WDQ) [41], which included a total of 17 items within the mentioned constructs, as noted in Table 2. In this part of the questionnaire, each item was rated on a five-point Likert scale, from “strongly disagree” (value 1) to “strongly agree” (value 5). Well-being was observed as the dependent variable through the Q-LES-Q-8 scale, a shortened version of the Q-LES-Q scale [42]. This questionnaire was designed to measure overall satisfaction by using statements that effectively captured the essential elements of well-being. This section contained a total of 12 statements that were evaluated using a five-point Likert scale, ranging from a value of 1 for “very poor” to a value of 5 for “very good”. The third section of the questionnaire collected information on the respondents’ characteristics. These statements were translated from English into Serbian and subsequently adapted to the research context. 

## 3. Data Analysis

The relationships between the constructs in this study underwent testing via the partial least squares structural equation modeling (PLS-SEM) approach. A confirmatory factor analysis (CFA) was conducted to determine whether the applied measurements aligned with the research constructs by launching the PLS-SEM algorithm. The results of the analysis pertaining to the assessment of reliability and validity are presented in Table 2.

The convergent validity, as measured with the average variance extracted (AVE), surpasses the threshold of 0.5 in all the instances, indicating that the constructs captured more than 50% of the variance in the items [43,44]. According to the results in Table 2, the AVE ranged from 0.554 to 0.829, meeting the required criteria. The composite reliability (CR) should exceed 0.7 [45], a condition that was satisfied in this research, indicating the strong internal consistency of the data. Multicollinearity is not a concern in this study, as evidenced by the variance inflation factor (VIF) being less than five [46]. Table 3 presents the results of the discriminant validity.

The discriminant validity assessment was conducted using the heterotrait–monotrait (HTMT0.85) criterion [47]. From the results in Table 3, the highest value observed was 0.799, while all the other values fell below it and were generally below the critical value of 0.850. Consequently, it can be concluded that the model exhibited satisfactory discriminant validity.

## 4. Results

The structural model estimation was performed using the PLSpredict algorithm, which included a cross-validated predictive ability test. The cross-validated redundancy index, also known as the Stone–Geisser Q^2^, was computed for the latent variable to assess its predictive significance. The value of the Stone–Geisser Q^2^ for OS was determined to be 0.318. Given the positive value of this indicator, it can be inferred that the structural model exhibited good quality [48,49].

The coefficient of determination (R^2^) suggested that 33% of the OS was explained by the model, indicating a relatively high level of explanatory power (Table 4). The standardized root mean square residual (SRMR) fell below the recommended threshold of 0.08 [50], indicating a good model fit. Additionally, the goodness-of-fit (GOF) value, which should ideally range between 0 and 1, was observed to be 0.324, falling within an acceptable range.

The standard procedure of PLS-SEM bootstrapping was employed to assess the value and significance of the path coefficients. This technique was utilized to compute 95 percent confidence intervals (CIs), which were corrected for bias on both sides, for all the relationships specified in the hypotheses. The findings of the structural model are summarized in Table 5. According to the results, KC exhibited a negative relationship with OS (β = −0.019; *p* > 0.05), but it was not statistically significant. Conversely, SC demonstrated a positive and statistically significant relationship with OS (β = 0.377; *p* < 0.001). Similarly, the relationship between TC and OS was positive, but there was an absence of statistical significance (β = 0.063; *p* > 0.05). Finally, the relationship between WC and OS was positive and statistically significant (β = 0.216; *p* < 0.001). The supported relationships between WC and OS and between SC and OS suggested a positive influence of work context and social characteristics as the aspects of work design on the determinants of perceived well-being. Specifically, a one-point increase in social characteristics led to an increase of 0.377 in the elements of overall satisfaction, while a one-point increase in work context resulted in an increase of 0.216 in this construct. The results of testing direct effects with multigroup partial least square path modeling are detailed in Table 6.

In the subsequent analysis, we conducted a comparative examination of the path coefficients among the distinct groups that were categorized according to two criteria: gender and age. Two groups were formed based on gender, while three groups were established based on age. In terms of the influence of KC on OS, there was a lack of statistical significance observed in both subsamples. Similar results were obtained when assessing the influence of TC on OS. However, regarding the impact of SC on OS, statistically significant results were observed in both females and males. Notably, this influence appeared to be slightly stronger in males (β = 0.600, *p* < 0.01). Conversely, the influence of WC on OS was found to be statistically significant only in females (β = 0.094, *p* < 0.01). These findings suggested that gender moderated the effects between SC and OS, as well as between WC and OS. When analyzing the influence of age, no statistically significant differences were evident in the relationship between KC and OS. However, significant differences were observed when examining the relationship between SC and OS. Specifically, statistical significance was found in all the age groups: individuals aged less than 40 (β = 0.345, *p* < 0.01), those with ages of 41–50 years (β = 0.416, *p* < 0.01), and those older than 51 years (β = 0.321, *p* < 0.01). Notably, the results were slightly more significant for the respondents aged 41–50 years, followed by the age group with less than 40 years and, finally, those older than 51 years. The influence of TC on OS was statistically significant only for the respondents under the age of 40 (β = 0.212, *p* < 0.05), but not for the other age groups. Likewise, a statistically significant difference in the impact of WC on OS was observed only in the respondents older than 51 years (β = 0.400, *p* < 0.001), with no significant effects being observed in the other age groups. These findings indicate that the relationships between SC and OS, TC and OS, and WC and OS varied depending on age.

## 5. Discussion

In general, the results indicated that certain components of work design can be significant predictors of the well-being of workers, a finding that was corroborated by previous studies [4]. Our research focused on the four aspects of job design, namely, task characteristics, knowledge characteristics, social characteristics, and the work context [41]. The analysis of particular relationships between the mentioned constructs and the perceived well-being of healthcare professionals revealed that the knowledge characteristics of work design do not have a statistically significant effect on well-being, although this was expected according to the job demand–resource theory [29]. However, the postulates of the mentioned theory were supported by the confirmation of the positive effect of social characteristics on perceived well-being [24,30]. The task characteristics of work design did not have a positive effect on well-being, although previous research has shown that interventions in this domain of work design can influence well-being [24,25]. The work context has a significant positive effect on healthcare professionals’ perceived well-being, supporting the findings of a prior study that emphasized the importance of the work environment for well-being [28].

The study’s results revealed a positive relationship between the social characteristics of work design and overall satisfaction. Pagán-Castaño et al. [7] emphasized that social relations constitute an important dimension of well-being, making them a determinant of positive mental states among employees in healthcare organizations [32]. In the work design within healthcare settings, it is imperative to consider the social dimension, which necessitates intensive communication, cooperation, and the cultivation of positive relationships among healthcare professionals. Establishing a supportive work environment where individuals feel valued and supported can significantly enhance their overall well-being. When colleagues and supervisors genuinely prioritize each other’s welfare, this fosters a sense of belonging and affinity. Both initiated and received interdependence in job roles can yield positive outcomes for individual well-being. When individuals perceive that their work directly influences the success of others and vice versa, this promotes teamwork and mutual reliance. This interdependence fosters collaboration, communication, and coordination among team members, leading to increased job satisfaction and a sense of collective achievement. Knowing that their contributions are integral to their team’s success can enhance individuals’ sense of purpose and accomplishment in their work. Constructive feedback from colleagues also plays a vital role. Job design that facilitates the exchange of feedback among employees is essential for professional growth and development. Furthermore, when individuals receive recognition and appreciation for their contributions, it boosts their self-esteem, motivation, overall job satisfaction, and well-being.

It is essential to note that the work context itself has a positive relationship with the overall satisfaction of the determinants of well-being. Consequently, work design in healthcare organizations should prioritize providing employees with tailored working conditions, essential equipment, and stimulating work environments. When employees have a comfortable workplace and proper support, they are less likely to experience physical discomfort or fatigue during their workday. Additionally, when employees perceive their physical capabilities as aligned with the demands of their jobs, they are more likely to feel competent and capable in their roles. When employees feel physically safe and protected in their work environment, they can focus on their tasks without distractions or concerns about their health and safety. The mentioned factors refer to the extrinsic nature of work design that should ensure well-being, as stated by Supardi et al. [19].

While the results revealed no statistically significant relationships between the knowledge characteristics of work design and the overall satisfaction of aspects of well-being, recording this outcome appears to be important due to one negative result. While learning and knowledge sharing are typically the intrinsic factors that positively impact well-being across various sectors [15,21], this phenomenon does not seem to hold within healthcare according to this research. According to Gordon et al. [24], healthcare organizations harbor numerous stress triggers that are capable of diminishing employee well-being. Healthcare professionals often contend with heavy workloads, extended work hours, stressful situations, and the pressing need to optimize healthcare quality despite limited resources. Given these challenges, healthcare workers may not prioritize minimizing their work complexity or fostering specialization through task division, which could otherwise enhance productivity. The demanding nature of healthcare work necessitates that task-related information is clearly delineated for employees to expedite decision making and knowledge creation. In other words, if healthcare professionals are burdened with independently formulating conclusions about tasks, problem-solving approaches, and required skills, their workload increases, leading to heightened physical and mental fatigue, thereby negatively impacting well-being. Elevated job complexity, information processing demands, and problem-solving requirements can elevate stress levels among healthcare professionals. The constant need to process vast amounts of information, solve complex problems, and apply specialized skills may contribute to the feelings of being overwhelmed, frustrated, and anxious. Consequently, this can diminish well-being and exacerbate feelings of burnout. Therefore, in designing healthcare workers’ tasks, it is imperative to provide comprehensive task-related information and requisite knowledge to maximize productivity and mitigate potential adverse employee reactions.

Although the relationship between task characteristics and overall satisfaction appeared to be positive, it was not statistically significant, contrary to findings in other studies [33,35]. This outcome can be interpreted in various ways. While autonomy is typically associated with positive effects on employee performance and behavior [28], a similar positive effect could be expected for task variety. However, it is important to consider that healthcare professionals often require clear guidelines and instructions for tasks to optimize healthcare delivery. Providing excessive freedom and introducing complex tasks may lead to ambiguity and confusion among employees, ultimately diminishing their well-being. While task complexity, variety, and significance can provide opportunities for skill development and engagement, they may also increase the cognitive and emotional demands on healthcare professionals. Complex tasks requiring high levels of decision-making autonomy and independence might lead to feelings of stress or pressure. Task identity, which involves completing a piece of work from beginning to end, can provide a sense of accomplishment. However, in the fast-paced and unpredictable environment of healthcare, interruptions, shifting priorities, and the need for continuous care may disrupt the completion of tasks. Healthcare professionals might struggle to experience the well-being associated with finishing tasks due to the transient and fragmented nature of their work. Therefore, managers and human resource professionals should exercise caution when defining autonomy, task variety, identity, and other task characteristics. 

In terms of multigroup effects, no statistically significant influence of knowledge characteristics on the overall satisfaction of the determinants of well-being was found among male and female healthcare professionals, as well as among employees in different age groups. This result confirms the earlier conclusion regarding the absence of a statistically significant relationship between knowledge characteristics and well-being. Regardless of gender and age, healthcare workers uniformly perceive knowledge characteristics as an aspect of work design, with an equal impact on their well-being. Regarding the influence of social characteristics on overall satisfaction, differences were observed between females and males, with this effect being more significant in females. According to Nowak and Mazurek [51], women tend to prioritize social values, harmony, cooperation, and interpersonal relationships more than men do, which could explain the heightened influence of social characteristics on well-being among females. When considering age, statistically significant results were observed across all the age groups, with the most noticeable effects being noted in the 41–50 age group. This finding is somewhat anticipated, as employees in this age range are typically at a pivotal stage in their careers, experiencing a surge in professional growth and responsibilities. At this stage, they may place greater emphasis on social characteristics, such as social support, collaboration, and interpersonal relationships in the workplace. Individuals in this age group often balance their career responsibilities with family obligations. As they navigate the demands of both work and family life, they may prioritize social characteristics that contribute to a supportive work environment. Social support from colleagues and supervisors, opportunities for flexible work arrangements, etc. play an important role in helping individuals in this age group manage work–life balance and cope with the complexities of their personal and professional lives. Considering the specific aspects of personal life within this age group and the imperative to fulfill private obligations, it is reasonable to assume that hedonic well-being is higher. 

The relationship between the task characteristics of work design and overall satisfaction did not exhibit statistically significant differences between females and males employed in healthcare organizations. This suggests that regardless of gender, they interpret the same degree of autonomy, task variety, and other work design characteristics in terms of their impact on well-being, as explained earlier. When comparing the results across the age groups, the impact on the youngest category of employees was statistically significant and distinct from the other age groups. This finding can be elucidated within the context of a study by Marques et al. [52]. For young employees, task variety emerges as a pertinent aspect of work design, as they seek to acquire additional knowledge and enhance existing skills. Task identity is significant in satisfying intrinsic needs for respect and self-actualization, driving younger employees towards this type of job design. Moreover, receiving feedback aids in better understanding their skills and performance, facilitating improvement. The influence of the work context on overall satisfaction is more significant for females than for males. This outcome can be understood in the context of the previously described influence of social characteristics, wherein women prioritize a working environment characterized by good social relations, collaboration, communication, and information sharing, leading to higher levels of well-being. Regarding age, there were differences primarily among the respondents who were over 51 years old. According to Shavit et al. [53], older employees favor working conditions featuring positive social relations among colleagues and opportunities for applying knowledge. As these employees transition into the later stages of their careers, the intrinsic factors of work design, such as those related to the work context, become increasingly important, contributing to higher levels of well-being in this demographic.

## 6. Conclusions

The aim of this study was to examine the relationship between the elements of work design and well-being among healthcare professionals. When used as a construct of work design, social characteristics demonstrated a significant positive impact on well-being, particularly among women and individuals aged 41–50 years. Notably, work context had a significant influence on well-being, especially when it came to females and the oldest age group in healthcare organizations. These results are important, as they provide insights into the complex dynamics of work design and well-being among healthcare professionals. This study’s findings revealed a negative relationship between the knowledge characteristics of work design and healthcare professionals’ well-being. Despite the intrinsic benefits of job complexity and specialization, the demanding nature of healthcare work may overshadow these advantages, leading healthcare professionals to prioritize clear task delineation and comprehensive task-related information to mitigate stress and fatigue. Finally, while the relationship between task characteristics and well-being appeared to be positive, it lacked statistical significance regarding gender and in nearly all the age groups, except for the youngest one. Understanding the relationships between the elements of work design and well-being can inform tailored interventions and strategies aimed at enhancing workplace environments and, ultimately, improving organizational outcomes.

## Figures and Tables

**Table 1:** Sample structure.

Variable	Frequency	Percentage
Gender		
Female	354	82.9%
Male	73	17.1%
Age		
<40	197	46.1%
41–50	114	26.7%
>51	116	27.2%
Education		
Graduates or higher	130	30.4%
High school degree or high school diploma	297	69.6%
In total	427	100%

**Table 2:** Measurement model and constructs.

Construct and Items	Loadings	VIF	α	CR	AVE
Work design determinants					
TCs: Task characteristics			0.898	0.910	0.829
TC01: The job allows me to plan how I do my work.	0.912	2.749			
TC02: The job provides me with significant autonomy in making decisions.	0.925	2.857			
TC03: The job allows me to make decisions about what methods I use to complete my work.	0.894	2.656			
KCs: Knowledge characteristics			0.861	0.896	0.590
KC01: The job requires that I engage in a large amount of thinking.	0.771	2.377			
KC02: The job requires me to keep track of more than one thing at a time.	0.708	2.156			
KC03: The job requires me to analyze a lot of information.	0.819	2.433			
KC04: The job often involves dealing with problems that I have not met before.	0.810	2.146			
KC05: The job requires a variety of skills.	0.704	1.620			
KC06: The job requires very specialized knowledge and skills.	0.790	2.027			
SCs: Social characteristics			0.827	0.831	0.659
SC01: I have the opportunity to develop close friendships in my job.	0.781	1.701			
SC02: My supervisor is concerned about the welfare of the people who work for him/her.	0.835	1.954			
SC03: People I work with take a personal interest in me.	0.829	1.791			
SC04: The job requires spending a great deal of time with people outside my organization.	0.801	1.683			
WC: Work context			0.750	0.785	0.554
SS01: The workplace allows for all size differences between people in terms of clearance, reach, eye height, leg room, etc.	0.793	1.332			
SS02: The workplace is free from excessive noise.	0.801	1.440			
SS03: The job has a low risk of accidents.	0.720	1.974			
SS04: The job occurs in a clean environment.	0.655	1.811			
Well-being determinants					
OS: Overall satisfaction with …			0.940	0.946	0.601
OS01: … physical health.	0.742	3.071			
OS02: … mood.	0.777	4.116			
OS03: … work.	0.776	2.696			
OS04: … household activities.	0.730	2.101			
OS05: … social relationships.	0.723	2.182			
OS06: … family relationships.	0.761	2.507			
OS07: … leisure-time activities.	0.793	2.613			
OS08: … ability to function in daily life.	0.748	2.242			
OS09: … sexual drive, interests, and/or performance.	0.769	2.479			
OS10: … ability to get around physically without feeling dizzy, unsteady, or falling.	0.832	3.375			
OS11: … vision in terms of ability to do work or hobbies.	0.833	3.172			
OS12: … overall sense of well-being.	0.806	2.784			

**Table 3:** Discriminant validity.

Constructs	1	2	3	4	5
1. KCs: Knowledge characteristics	–				
2. OS: Overall satisfaction	0.324				
3. SCs: Social characteristics	0.547	0.612			
4. TCs: Task characteristics	0.520	0.422	0.732		
5. WC: Work context	0.746	0.525	0.799	0.621	–

**Table 4:** Fit indices of the structural model.

Construct	Stoner–Geisser Q^2^	R^2^	GOF
Overall satisfaction	0.318	0.330	0.324
SRMR	0.078		

**Table 5:** Results of testing direct effects.

Relationship	Path Coefficient	*t*-Value	95% CIs (Bias-Corrected)	Results
KCs → OS	−0.019	0.384	[−0.119, 0.077]	Not supported
SCs → OS	0.377 ***	5.848	[0.248, 0.500]	Supported
TCs → OS	0.063	1.101	[−0.044, 0.181]	Not supported
WC → OS	0.216 ***	3.630	[0.097, 0.328]	Supported

Notes: KCs, knowledge characteristics; SCs, social characteristics; TCs, task characteristics; WC, work context; OS, overall satisfaction; *** *p* < 0.001.

**Table 6:** Results of testing direct effects: multigroup partial least square path modeling.

Relationship	Path Coefficient	*p*-Value	Path Coefficient	*p*-Value	Path Coefficient	*p*-Value	Invariant
	Female	Female	Male	Male			
KCs → OS	0.011	0.843	−0.057	0.692			Yes
SCs → OS	0.342	0.000 ***	0.600	0.001 **			Yes
TCs → OS	0.094	0.140	−0.171	0.266			Yes
WC → OS	0.219	0.001 **	0.179	0.179			No
	Age < 40	Age < 40	Age 41–50	Age 41–50	Age > 51	Age > 51	
KCs → OS	−0.037	0.673	0.052	0.550	−0.011	0.898	Yes
SCs → OS	0.345	0.003 **	0.416	0.001 **	0.321	0.002 **	Yes
TCs → OS	0.212	0.033 *	0.021	0.819	−0.100	0.341	No
WC → OS	0.150	0.109	0.147	0.199	0.400	0.000 ***	No

Notes: KCs, knowledge characteristics; SCs, social characteristics; TCs, task characteristics; WC, work context; OS, overall satisfaction. * *p* < 0.05; ** *p* < 0.01; *** *p* < 0.001.

## Data Availability

The data that support the findings of this study are available from the corresponding author upon reasonable request.
